# Cut-off values of Geriatric Nutritional Risk Index for cardiovascular events in Japanese patients with acute myocardial infarction

**DOI:** 10.1007/s00380-024-02455-w

**Published:** 2024-09-13

**Authors:** Satoshi Ito, Yasunori Inoue, Tomohisa Nagoshi, Takatoku Aizawa, Yusuke Kashiwagi, Satoshi Morimoto, Kazuo Ogawa, Kosuke Minai, Takayuki Ogawa, Michihiro Yoshimura

**Affiliations:** Division of Cardiology, Department of Internal Medicine, The Jikei University School of Medicine, 3-25-8 Nishi-Shinbashi, Minato-ku, Tokyo, 105-8461 Japan

**Keywords:** Ischemic heart disease, Nutrition, Time-dependent receiver operating characteristic analysis, Prognosis

## Abstract

The Geriatric Nutritional Risk Index (GNRI) is a straightforward nutritional risk assessment tool with an established relationship with poor prognosis in patients with heart failure. However, the utility of the GNRI in patients with acute myocardial infarction (AMI) remains unclear given the time-dependent changes in the pathophysiology of AMI and the selected endpoints. Accordingly, we aimed to evaluate the optimal cut-off values of the GNRI for cardiovascular events in patients with AMI. We used time-dependent receiver operating characteristic analysis to identify the optimal cut-off values for two endpoints, all-cause death and major adverse cardiac events (MACE: all-cause death, non-fatal myocardial infarction, hospitalization for heart failure, and stroke), over 4 years in 360 patients with AMI between 2012 and 2020. The cumulative incidence of MACE was 11.6%. The cut-off value of the GNRI for all-cause death was 82.7 (area under the curve [AUC], 0.834) at 3 months and 90.3 (AUC 0.854) at 4 years. The cut-off value of the GNRI for MACE was 83.0 (AUC 0.841) at 3 months and 95.3 (AUC 0.821) at 4 years. The GNRI demonstrated consistently high reliability relative to other indicators of AMI. Our findings indicated that the optimal cut-off value and reliability of the GNRI for cardiovascular events varied according to the endpoints and observation periods. GNRI emerges as a crucial predictor of prognosis for patients with AMI.

**Supplementary Information:**

The online version contains supplementary material available at 10.1007/s00380-024-02455-w.

## Introduction

The Geriatric Nutritional Risk Index (GNRI), which is based on serum albumin levels and body mass index (BMI), is a simple nutritional risk assessment tool. The GNRI is used to assess the nutritional status of patients with various diseases and has been reported to be associated with adverse events. Specifically, a low GNRI in patients with cancer has been reported to be associated with poor prognosis [1–5]. Moreover, the significance of the GNRI in cardiovascular diseases appears to be substantial. There have been numerous reports on the relationship between heart failure and the GNRI, indicating a considerable body of evidence in this regard [6–10].

On the other hand, when examining previous studies on ischemic heart disease (IHD), there are somewhat divergent perspectives. Some reports have suggested the utility of the GNRI in patients with IHD [11–13]. Moreover, malnutrition assessed using the GNRI is an independent predictor of long-term mortality in patients with acute myocardial infarction (AMI) [14]. Meanwhile, although the GNRI has not been shown as an independent predictor of major adverse cardiac and cerebrovascular events, malnutrition itself has been strongly associated with adverse outcomes in older patients with AMI [15]. However, the impact of GNRI in patients with AMI remains unclear and requires further study. In addition, it may be necessary to consider the use of the GNRI in IHD separately with respect to chronic and acute conditions. Specifically, in acute myocardial infarction (AMI), it is important to consider the time-dependent significance of the GNRI; further, the utility of the GNRI may differ according to the selected endpoints.

Receiver operating characteristic (ROC) curves are commonly used to evaluate the cut-off values and efficacy for the occurrence of endpoint events. Previous studies on the cut-off values for the GNRI have applied the standard ROC analysis. However, despite the importance of the time course in disease outcomes, the standard ROC analysis does not consider the time parameter, which is a crucial factor to consider with respect to the time course of the incidence of cardiovascular events. Contrastingly, time-dependent ROC analysis can account for dropout cases during the follow-up period and evaluate predictive ability using the area under the curve (AUC). Several studies have investigated prognosis using time-dependent ROC analyses [16–20] and demonstrated the appropriateness of evaluating the ROC curve as a function of time. Thus, the relevance of time-dependent ROC analysis methods should be reestablished for future research. This study aimed to investigate the optimal cut-off values of the GNRI in the short and long term for two endpoints in patients with AMI using time-dependent ROC analysis.

## Materials and methods

### Study patients

We included patients with AMI who required emergency admission to the Jikei University Hospital between January 7, 2012, and February 21, 2020. We last accessed the study data on September 11, 2021, for research purposes. Patients were followed for up to 4 years. A diagnosis of AMI was indicated by the presence of any two of the following three criteria: (1) cardiac chest pain lasting for ≥ 30 min, (2) new or presumed new significant ST-T segment changes, and (3) an increase in cardiac biomarker levels (serum creatine kinase or cardiac troponin I) [21]. All patients were admitted to the hospital and underwent emergent cardiac catheterization. During the study period, all patients were treated for AMI. We defined the day of admission as the day 1. We excluded the patients who were lost to follow-up after discharge (*n* = 32). Based on these selection criteria, we enrolled 360 consecutive patients.

The study protocol was approved by the ethics committee of the Jikei University School of Medicine (27-103[7988]), and we complied with the routine ethical regulations of our institution. All clinical investigations were conducted in accordance with the principles expressed in the Declaration of Helsinki. General informed consent and notification are provided on admission for all studies performed in our hospital. Although a specific informed consent for this study was not obtained for each patient because this was a retrospective study, we posted a notice about the study design and contact information in a public location in our institution according to our routine ethical regulations. In this public notification, we ensured that patients had the opportunity to refuse to participate (opt-out) in the study. The researchers were only given access to a database containing completely anonymous information.

### Data collection

The clinical characteristics were retrospectively collected from the hospital medical records. Biochemical tests, including measurement of serum albumin levels, were performed at the time of the emergent catheterization. The hemodynamic parameters were measured during cardiac catheterization.

### Measurements and assessments of GNRI

Nutritional status was assessed using the GNRI as defined by Bouillanne et al. [22].

The GNRI was calculated using the serum albumin levels and BMI at admission, as reported by Yamada et al. [23]. A BMI (kg/m^2^) of 22 was defined as the ideal weight; if the patient’s BMI exceeded 22, the ratio of weight to ideal weight was set to 1.

$$\text{GNRI}=14.89 \times \text{serum albumin} (\text{g}/\text{dL})+41.7\times (\text{BMI}\div 22 \text{kg}/\text{m}2).$$

### Study endpoint

Two endpoints were considered in this study. The first endpoint was defined as all-cause death, while the second endpoint was defined as major adverse cardiac events ([MACE]; all-cause death, non-fatal myocardial infarction, hospitalization for heart failure, and stroke). We evaluated the incidence of endpoints at 3 months, 6 months, 1 year, 2 years, 3 years, and 4 years after hospitalization for AMI.

### Statistical analysis

Continuous variables are expressed as the means ± standard deviation (SD) or the median of the relevant range. Categorical variables are expressed as percentages. The incidence of endpoints after hospitalization for AMI is shown using Kaplan–Meier curves. *P* < 0.05 was considered statistically significant. Statistical analyses were performed using SPSS Statistics software (version 22.0; IBM Corp., Armonk, NY, USA).

In this study, we applied time-dependent ROC analysis. ROC analysis is a well-established statistical method for evaluating the strength of the correlation between the independent variable, which is a continuous variable, and the outcome, which is a dichotomous variable. However, the ROC curve assumes that disease outcome does not change over time. However, in practice, disease outcomes are time dependent given the temporal changes in status. Therefore, time-dependent ROC analysis was used to evaluate the predictive utility of the independent variables for time-dependent disease outcomes [24].

Individual disease outcomes were observed and updated at each time point in the time-dependent ROC analysis. The Youden index was used to calculate the optimal cut-off value. Statistical analysis of the time-dependent ROC was performed using EZR (Saitama Medical Center, Jichi Medical University, Saitama, Japan), which is a graphical user interface for R (The R Foundation for Statistical Computing, Vienna, Austria) [25]. EZR is a modified version of the R commander, which is designed to add statistical functions that are frequently used in biostatistics. We used the “survival ROC” package, written for R, to assess the optimal cut-off values of the GNRI in the short and long term for the two endpoints using time-dependent ROC analysis.

## Results

### Clinical characteristics of the study patients

Table 1 shows the clinical characteristics of the included patients. The mean age of the patients was 62 ± 13 years. Among the patients, 88.9% were male. The mean GNRI was 97.9 ± 8.3. A total of 268 (74.4%) and 92 (25.6%) patients were diagnosed with ST-segment and non-ST-segment elevation myocardial infarction, respectively. Moreover, 34.7% and 67.5% of the patients were diagnosed with diabetes mellitus and hypertension, respectively, while 6.1% were undergoing hemodialysis. The mean left ventricular ejection fraction (LVEF) was 50.6 ± 10.5%. Renin–angiotensin–aldosterone system inhibitors, *β*-blockers, and insulin were prescribed to 32.8%, 15.0%, and 6.1% of the patients prior to hospital admission, respectively.

**Table 1:** Clinical characteristics on admission (*n* = 360)

Characteristic	Mean ± SD or median [interquartile range]
Age, years	62 ± 13
Sex, male (%)	320 (88.9)
BMI, kg/m^2^	25.1 ± 4.2
Hb, g/dL	13.6 ± 2.1
Alb, g/dL	3.8 ± 0.5
Cr, mg/dL	0.8 [0.7–1.0]
eGFR, mL/min/1.73 m^2^	70.0 ± 27.2
UA, mg/dL	5.9 ± 1.6
Glucose, mg/dL	168.8 ± 77.2
HbA1c (%)	5.9 [5.6–6.7]
TG, mg/dL	72.0 [46.0–127.8]
HDL-C, mg/dL	49.2 ± 13.3
LDL-C, mg/dL	121.5 ± 37.3
BNP, pg/mL	46.1 [14.4–142.1]
LVEF, %	50.6 ± 10.5
GNRI	97.9 ± 8.3
*Diagnosis*	
STEMI (%)	268 (74.4)
NSTEMI (%)	92 (25.6)
*Comorbidities*	
Old myocardial infarction (%)	31 (8.6)
Diabetes mellitus (%)	125 (34.7)
Hypertension (%)	243 (67.5)
Dyslipidemia (%)	261 (72.5)
Hemodialysis (%)	22 (6.1)
Cancer carrier (%)	35 (9.7)
*Medication prior to admission (%)*	
Antiplatelet therapy	86 (23.9)
Beta-blocker	54 (15.0)
RAAS-I	118 (32.8)
Statin	91 (25.3)
Oral hypoglycemia agent	65 (18.1)
Insulin	22 (6.1)
Diuretics	20 (5.6)

SD, standard deviation; BMI, body mass index; Hb, hemoglobin; Alb, albumin; Cr, creatinine; eGFR, estimated glomerular filtration rate; UA, uric acid; HbA1c, glycated hemoglobin; TG, triglycerides; HDL-C, high-density lipoprotein; LDL-C, low-density lipoprotein; BNP, B-type natriuretic peptide; LVEF, left ventricular ejection fraction; GNRI, Geriatric Nutritional Risk Index; STEMI, ST-segment elevation myocardial infarction; NSTEMI, non-ST-segment elevation myocardial infarction; RAAS, renin–angiotensin–aldosterone system inhibitors

### Cumulative incidence of all-cause death and MACE

**Fig. 1:**
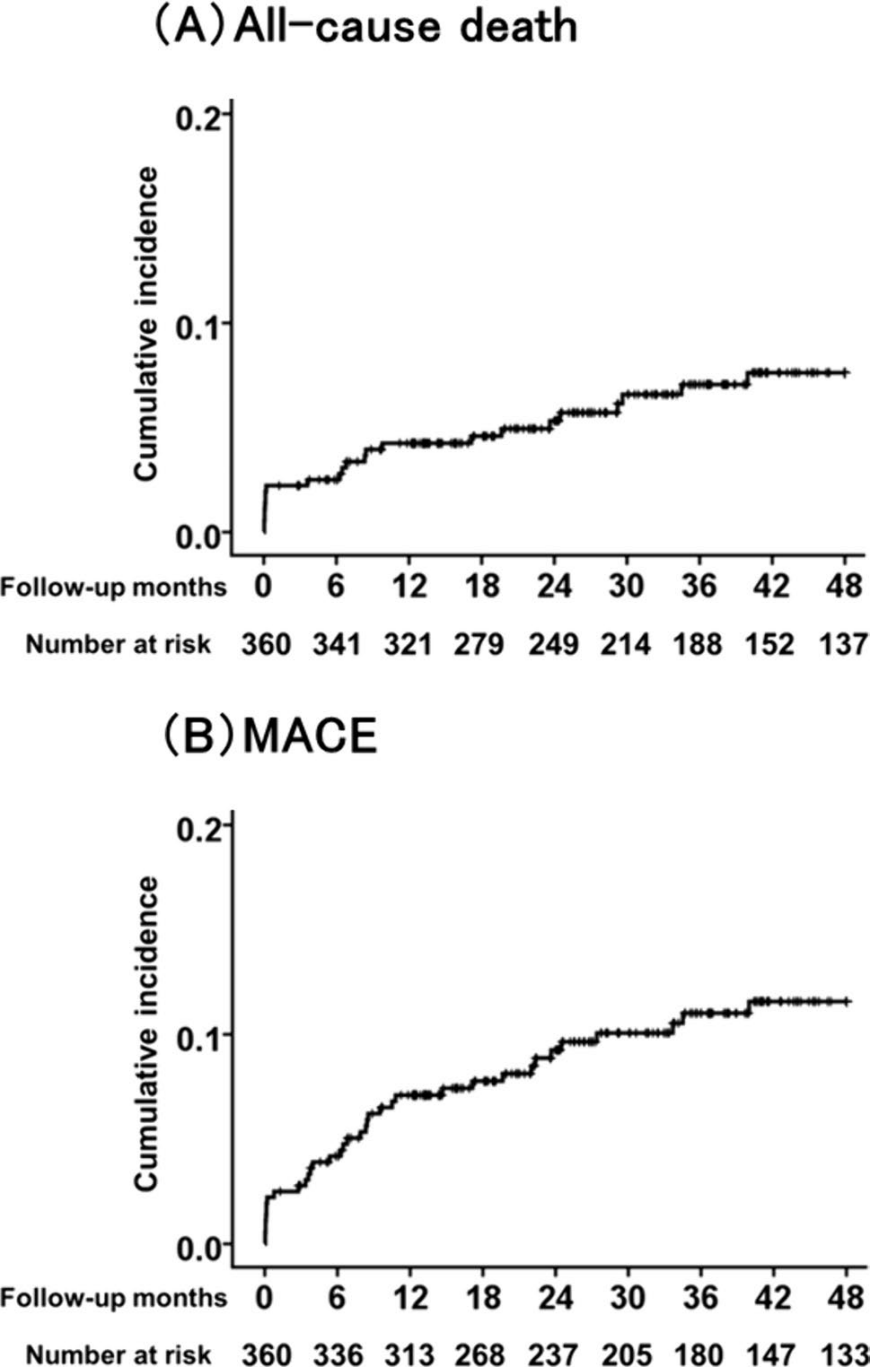
Cumulative incidence of all-cause death and MACE. **A** The cumulative incidence rates of all-cause death were 2.2%, 2.5%, 4.2%, 5.3%, 7.1%, and 7.6% at 3 months, 6 months, 1 year, 2 years, 3 years, and 4 years, respectively. **B** The cumulative incidence rates of MACE were 2.8%, 4.2%, 7.1%, 9.3%, 11.0%, and 11.6% at 3 months, 6 months, 1 year, 2 years, 3 years, and 4 years, respectively. MACE, major adverse cardiac events

During the observation period, 23 deaths, 3 non-fatal myocardial infarctions, 11 hospitalizations for heart failure, and 2 strokes occurred. The median follow-up period for all-cause death was 1103 (571–1440) days. The cumulative incidence rates of all-cause death were 2.2%, 2.5%, 4.2%, 5.3%, 7.1%, and 7.6% at 3 months, 6 months, 1 year, 2 years, 3 years, and 4 years, respectively (Fig. 1A). The cumulative incidence rates of MACE were 2.8%, 4.2%, 7.1%, 9.3%, 11.0%, and 11.6% at 3 months, 6 months, 1 year, 2 years, 3 years, and 4 years, respectively (Fig. 1B).

### Results of time-dependent ROC analysis

**Fig. 2:**
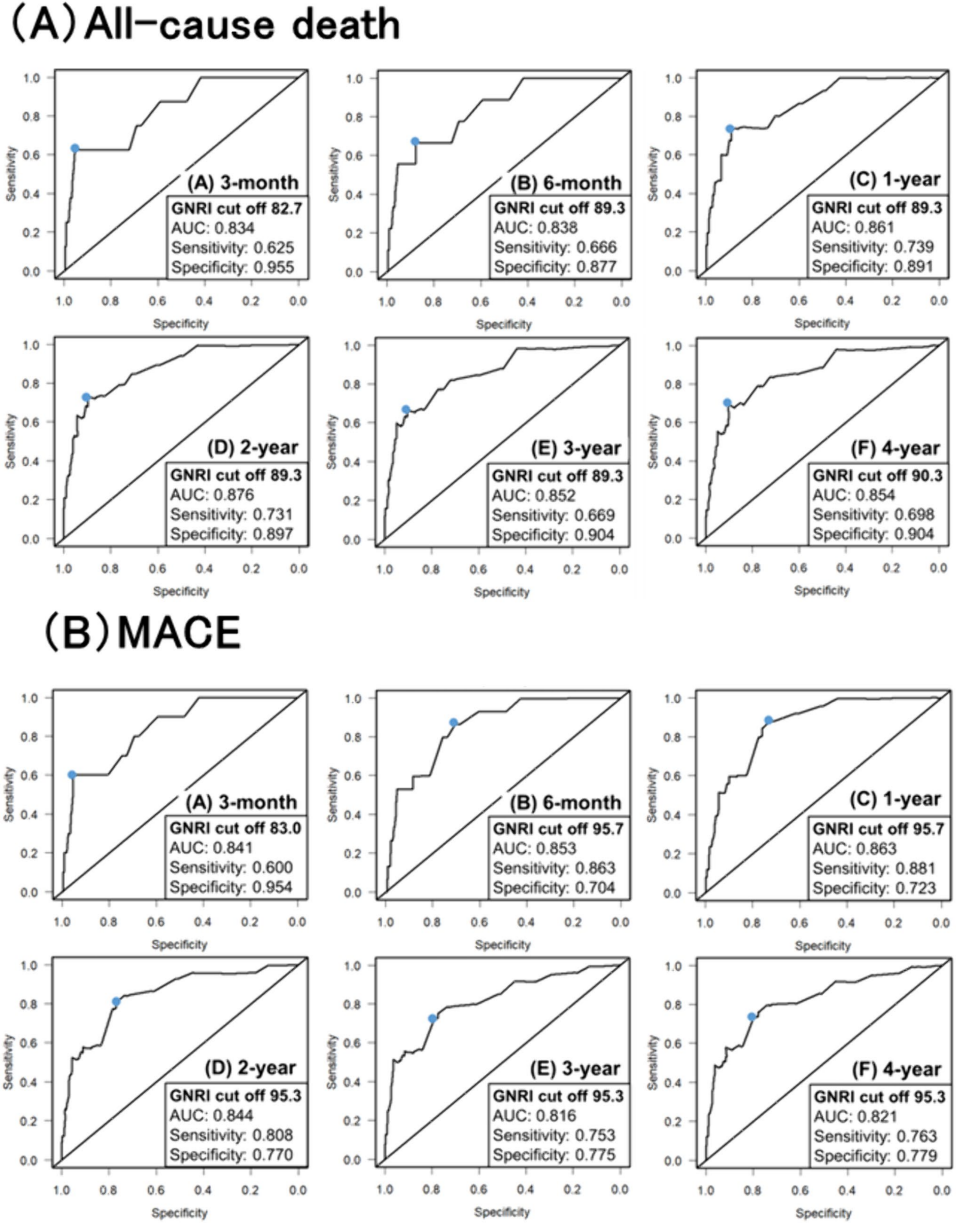
Time-dependent ROC analysis of GNRI for all-cause death and MACE. **A** Time-dependent ROC curve analysis of all-cause death at (A) 3 months, (B) 6 months, (C) 1 year, (D) 2 years, (E) 3 years, and (F) 4 years. **B** Time-dependent ROC analysis of MACE at (A) 3 months, (B) 6 months, (C) 1 year, (D) 2 years, (E) 3 years, and (F) 4 years. AUC, area under the curve; GNRI, Geriatric Nutritional Risk Index; MACE, major adverse cardiac event; ROC, receiver operating characteristic

The time-dependent ROC curve for all-cause death between 3 months and 4 years is shown in Fig. 2A. The optimal cut-off value of the GNRI for all-cause death was 82.7 in the short term (3 months), which was the lowest value among all the observation periods. Thereafter, the optimal cut-off value of the GNRI remained constant at 89.3 from 6 months to 3 years and slightly increased to 90.3 at 4 years. Figure 2B shows the time-dependent ROC curve for MACE from 3 months to 4 years. The optimal cut-off value of the GNRI was 83.0 in the short term (3-month), which was the lowest value among all the observation periods. Subsequently, the optimal cut-off value of the GNRI was almost constant at 95 after 6 months. The optimal cut-off values of the GNRI for the two endpoints were low and high in the short and long term, respectively. The optimal cut-off values of the GNRI for all-cause death tended to be lower than those for MACE during the observation period.

**Fig. 3:**
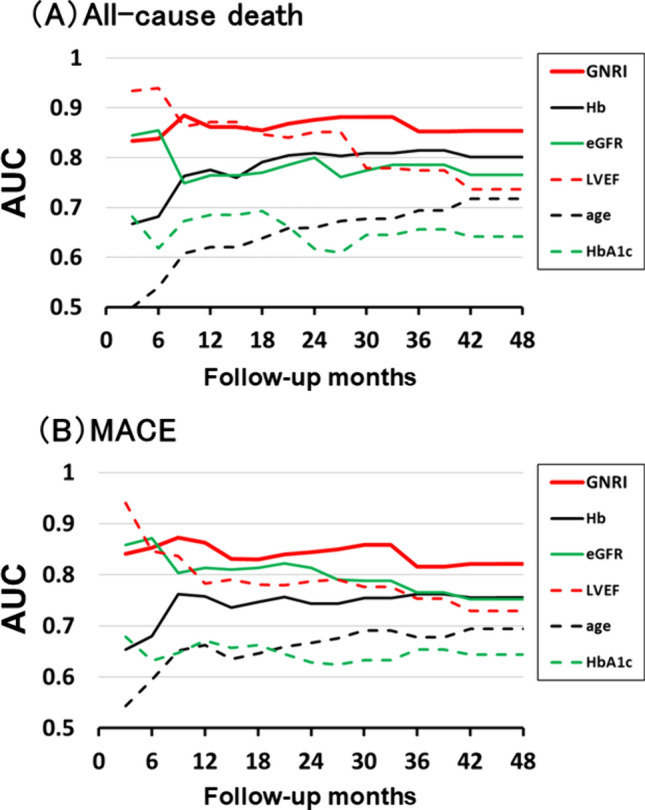
Changes over time in the AUC for all-cause death and MACE determined through time-dependent ROC analysis. **A** This figure shows the AUC values of several markers (GNRI, Hb, eGFR, LVEF, age, and HbA1c) for all-cause death from the start of follow-up. **B** This figure shows the AUC values of several markers (GNRI, Hb, eGFR, LVEF, age, and HbA1c) for MACE from the start of follow-up. ROC, receiver operating characteristic; GNRI, Geriatric Nutritional Risk Index; Hb, hemoglobin; eGFR, estimated glomerular filtration rate; LVEF, left ventricular ejection fraction; HbA1c, glycated hemoglobin; MACE, major adverse cardiac events; AUC, area under the curve

Changes in the AUC values for all-cause death over time from the start of follow-up by time-dependent ROC analysis are shown in Fig. 3A. Specifically, this figure shows the AUC values of several markers (GNRI, hemoglobin [Hb], estimated glomerular filtration rate [eGFR], LVEF, age, and glycated hemoglobin [HbA1c]) that might be associated with the prognosis of patients with AMI. The AUC values of the GNRI for all-cause death determined through time-dependent ROC analysis were 0.834, 0.838, 0.861, 0.876, 0.852, and 0.854 at 3 months, 6 months, 1 year, 2 years, 3 years, and 4 years, respectively. Each factor was strongly associated with all-cause death in patients with AMI. The AUC value of the GNRI for all-cause death tended to be high during the observation period and was not inferior to those of the other factors.

Changes in the AUC values for MACEs over time from the start of follow-up by time-dependent ROC analysis are shown in Fig. 3B. Specifically, this figure shows the AUC values of several markers that might be associated with AMI prognosis. The AUC values of the GNRI for MACE determined through time-dependent ROC analysis were 0.841, 0.853, 0.863, 0.844, 0.816, and 0.821 at 3 months, 6 months, 1 year, 2 years, 3 years, and 4 years, respectively. Each factor was strongly associated with MACE in patients with AMI. The AUC value of the GNRI for MACE tended to be high during the observation period and was not inferior to those of the other factors.

**Fig. 4:**
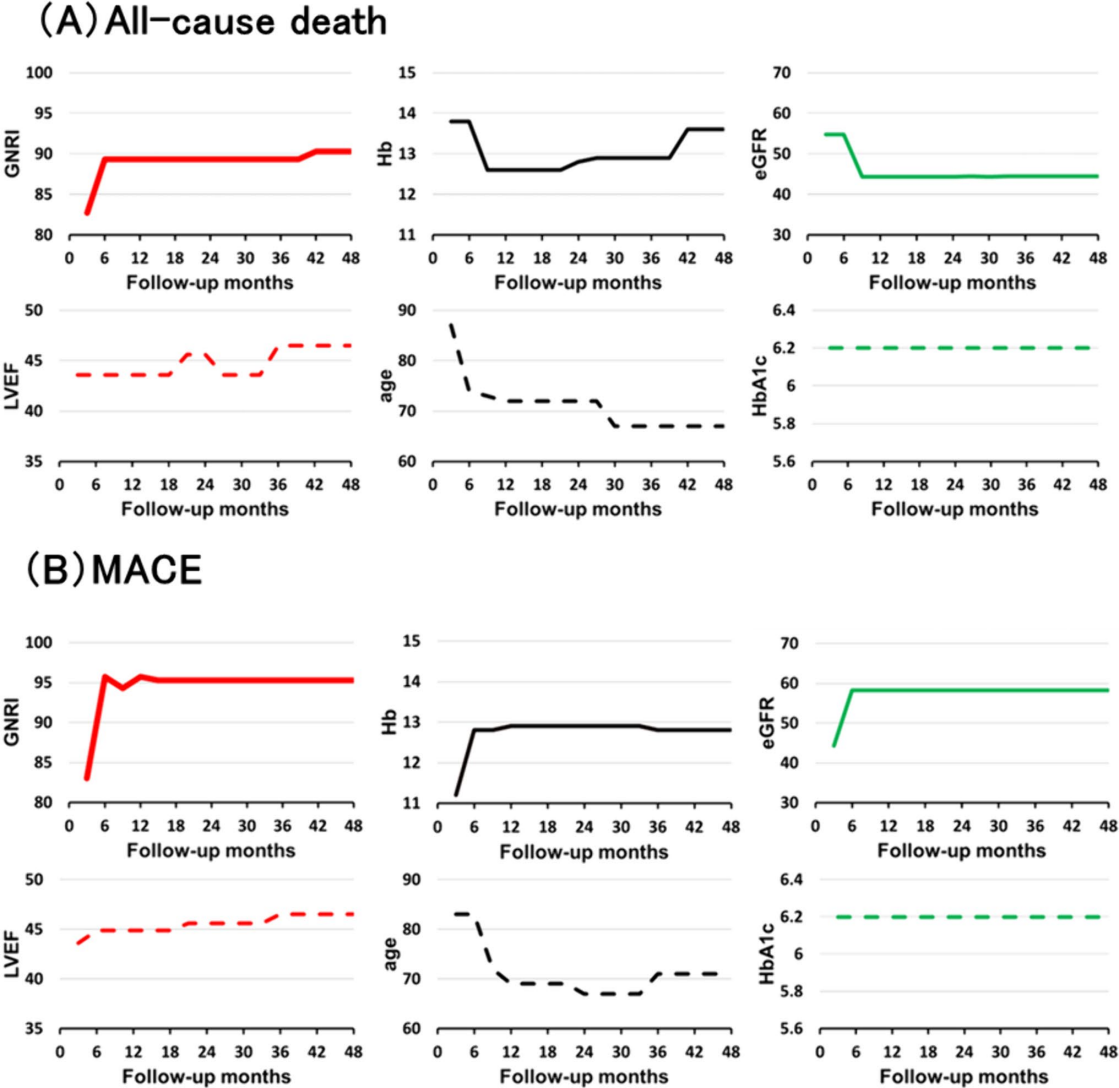
Changes over time in the cut-off value for all-cause death and MACE determined through time-dependent ROC analysis. **A** This figure shows the changes over time in the cut-off value of several markers (GNRI, Hb, eGFR, LVEF, age, and HbA1c) for all-cause death from the start of follow-up. **B** This figure shows the changes over time in the cut-off value of several markers (GNRI, Hb, eGFR, LVEF, age, and HbA1c) for MACE from the start of follow-up. ROC, receiver operating characteristic; GNRI, Geriatric Nutritional Risk Index; Hb, hemoglobin; eGFR, estimated glomerular filtration rate; LVEF, left ventricular ejection fraction; HbA1c, glycated hemoglobin; MACE, major adverse cardiac events

Figure 4A shows the changes over time in the cut-off values of several markers (GNRI, Hb, eGFR, LVEF, age, and HbA1c) for all-cause death from the start of follow-up. The optimal cut-off value of the GNRI for all-cause death was low in the short term but high in the long term. Figure 4B shows the changes over time in the cut-off values of several markers for MACE from the start of the follow-up. The optimal cut-off value of the GNRI for MACE was low in the short term but high in the long term. Thus, the optimal cut-off values for the GNRI varied across the endpoints.

Moreover, we evaluated changes over time in the AUC values for all-cause death and MACE determined through time-dependent ROC analysis, excluding patients undergoing hemodialysis (Fig. S1). Specifically, this figure shows the AUC values of several markers (GNRI, Hb, eGFR, LVEF, age, and HbA1c) that might be associated with the prognosis of patients with AMI. Even in this patient subgroup, the AUC of the GNRI for all-cause death and MACE tended to be high during the observation period and was not inferior to those of the other factors. Figure S2 shows the temporal changes in the cut-off values of several markers for all-cause death and MACE from the start of follow-up, with exclusion of patients undergoing hemodialysis. In this patient subgroup, the cut-off values of the GNRI were high in the long term and varied across the endpoints.

### Internal bootstrap validation of GNRI

**Fig. 5:**
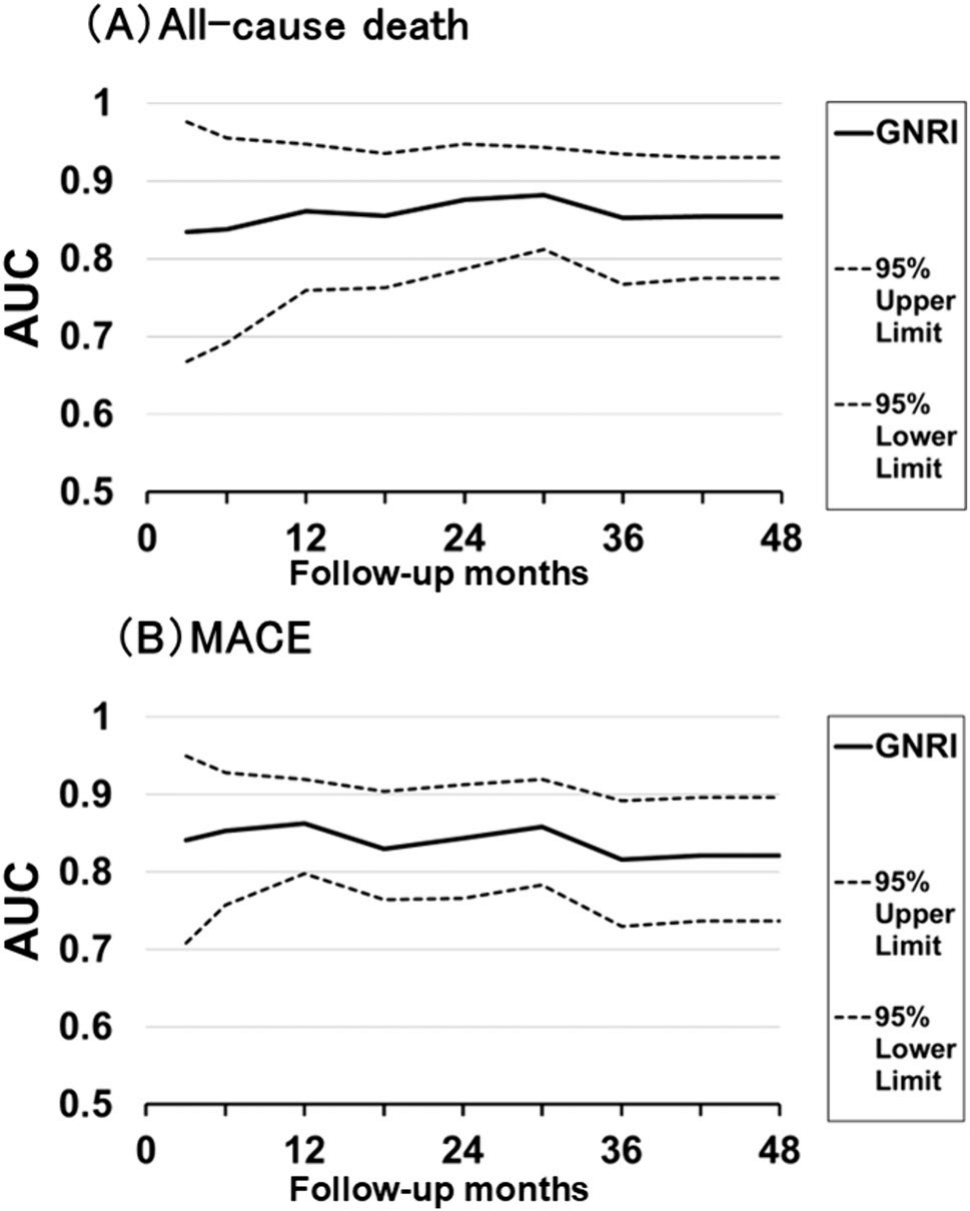
Changes over time in the AUC (95% CI) values of GNRI for predicting all-cause death (**A**) and MACE (**B**), obtained using internal bootstrap validation based on 1000 simulations. ROC, receiver operating characteristic; GNRI, Geriatric Nutritional Risk Index; MACE, major adverse cardiac event; AUC, area under the curve; CI, confidence interval

This study used internal bootstrap validation based on 1,000 simulations to evaluate the utility of the GNRI as a prognostic predictor in patients with AMI. The results of this validation method are presented in Fig. 5. Figure 5A shows the AUC values (95% confidence intervals) of the GNRI for all-cause death, derived through time-dependent ROC analysis, which were 0.834 (0.668–0.976) in the short term (3 months) and 0.854 (0.775–0.930) in the long term (4 years). Figure 5B shows the corresponding values for MACE determined through time-dependent ROC analysis, which were 0.841 (0.708–0.950) and 0.821 (0.737–0.896), respectively. The AUC values of the GNRI for both endpoints were high in the short- and long term. Overall, this analysis revealed that the GNRI has high prognostic value for short- and long-term cardiovascular outcomes in patients with AMI.

### Clinical outcomes with the optimal cut-off values of GNRI

**Fig. 6:**
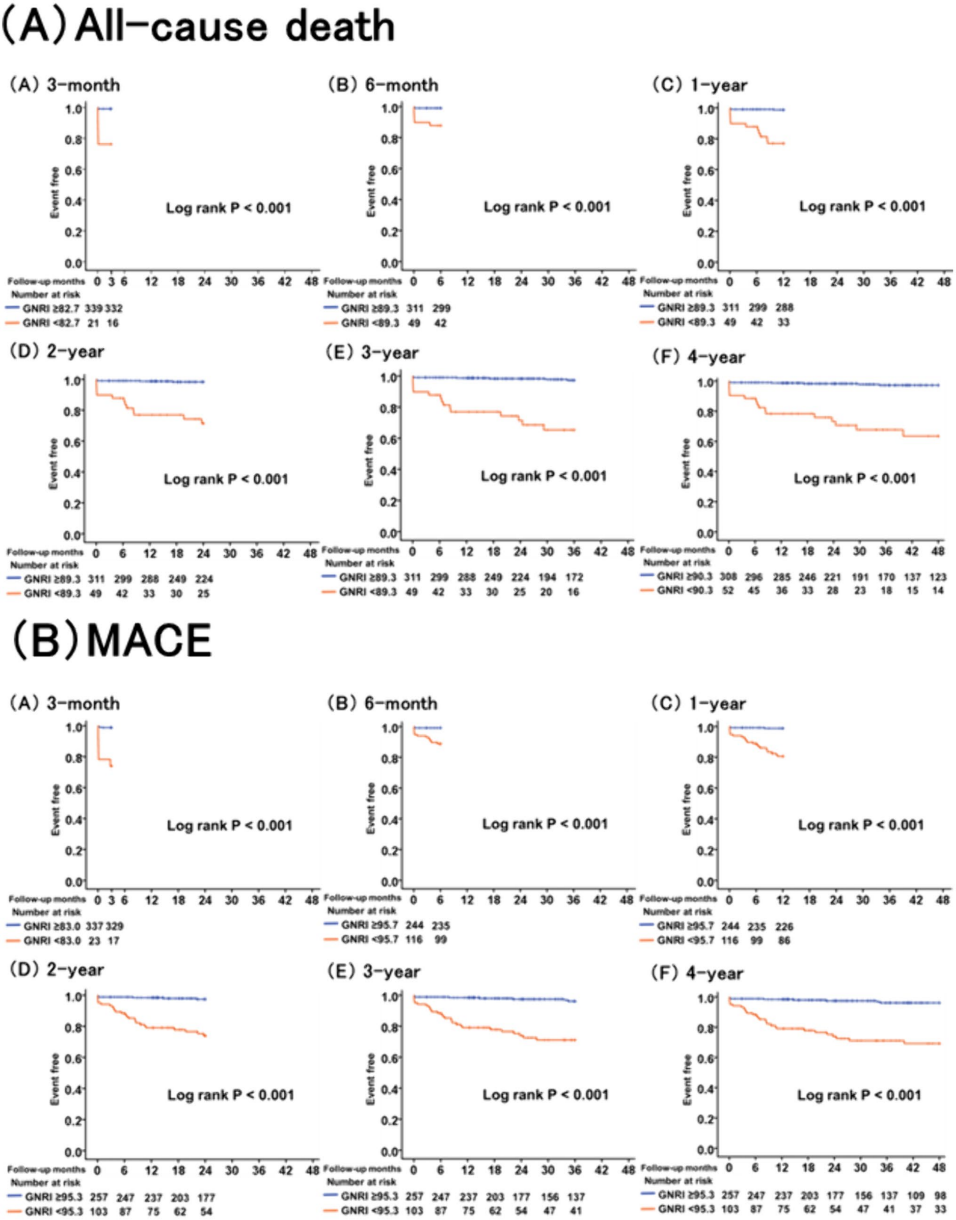
Kaplan–Meier curve for all-cause death and MACE in two groups divided by the optimal cut-off values of GNRI. **A** Kaplan–Meier curve for all-cause death at (A) 3 months, (B) 6 months, (C) 1 year, (D) 2 years, (E) 3 years, and (F) 4 years. **B** Kaplan–Meier curve for MACE at (A) 3 months, (B) 6 months, (C) 1 year, (D) 2 years, (E) 3 years, and (F) 4 years. GNRI, Geriatric Nutritional Risk Index; MACE, major adverse cardiac events

Kaplan–Meier curves were used to show the incidence of all-cause death in two groups divided by the optimal cut-off value of the GNRI at 3 months, 6 months, 1 year, 2 years, 3 years, and 4 years after hospitalization for AMI (Fig. 6A). The incidence of all-cause death was significantly higher in the group below the optimal cut-off value of the GNRI than in that above the optimal cut-off value of the GNRI during the observation period (*P* < 0.001).

Moreover, Kaplan–Meier curves were used to show the incidence of MACE in two groups divided by the optimal cut-off value of the GNRI (Fig. 6B). The incidence of MACE was significantly higher in the group below the optimal cut-off value of the GNRI than in that above the optimal cut-off value of the GNRI during the observation period (*P* < 0.001).

## Discussion

### Summary of findings

Our findings indicated that GNRI is a crucial and non-inferior indicator compared with the other indicators of AMI. Moreover, the AUC value of the GNRI determined through time-dependent ROC analysis was consistently high throughout the observation period, indicating the high reliability of the GNRI. In this study, we designed two endpoints. Although the endpoints changed, the GNRI was important in both cases. Moreover, the optimal cut-off values of the GNRI varied with each endpoint; specifically, the optimal cut-off values of the GNRI for the two endpoints were low in the short term but high in the long term.

### Comparison with other factors

We compared the significance of the GNRI with that of the other factors. Our findings indicated that the significance of the GNRI is comparable to that of Hb, eGFR, LVEF, age, and HbA1c. This holds true whether viewed in chronological order or by altering the outcome events. However, our statistical analysis has limitations and does not suggest that the GNRI is the most crucial among these factors. It would be more accurate to state that the GNRI is not inferior to the other indicators.

### Comparison with previous reports on AMI

In this study, the optimal cut-off value of the GNRI for all-cause death was 82.7 in the short term, increasing to 90.3 in the long term. In addition, the optimal cut-off value of the GNRI for MACE was 83.0 in the short term, and it remained almost constant at 95 in the long term. Regarding previous international reports, Kim et al. reported that the cut-off value of the GNRI for all-cause death in patients with AMI was 105 during a 1-year follow-up period [26]. Moreover, Jia et al. found that patients with AMI in the low GNRI group (< 98) had a significantly higher incidence of cardiac death than those in the high GNRI group (> 98) during a median follow-up period of 12.4 months [27]. Similarly, Kaplan et al. reported that the cut-off values of the GNRI for all-cause death and repeated percutaneous coronary intervention in patients with AMI were 90.68 and 94.55, respectively, during a 3-year follow-up period [28]. Contrastingly, among Japanese studies, Kanda reported that the cut-off value of the GNRI for all-cause death in patients with AMI was 90 within 30 days and 103 over 30 days [29]. Moreover, Abe et al. found that among patients with AMI, patients with a low GNRI (< 98) on arrival had a significantly higher incidence of all-cause death and primary composite endpoints (all-cause death, non-fatal stroke, non-fatal AMI, and hospitalization for heart failure) than patients with a high GNRI (> 98) during a median follow-up duration of 39 months [30]. Despite these variations, the results of the present study generally align with previous studies, indicating consistency in findings. Moreover, despite there being few reports, there may not be significant racial differences.

The reason for the lack of demonstrated utility of the GNRI in previous studies on ischemia may be attributed to potential between-study differences in settings, including the configuration of the GNRI values, study duration, and event specifications. It is conceivable that if settings similar to those adopted in our study were implemented, there is a possibility that the effectiveness of the GNRI could be demonstrated.

### Prognostic value of GNRI compared to albumin and BMI

We examined the prognostic values of the GNRI for all-cause death and MACE in patients with AMI and compared them to those of albumin levels and BMI, using the C-index. The results for all-cause death were 0.857 (95% CI 0.786–0.928), 0.869 (95% CI 0.802–0.936), and 0.588 (95% CI 0.460–0.715), respectively; the corresponding values for MACE were 0.827 (95% CI 0.762–0.892), 0.834 (95% CI 0.771–0.897), and 0.549 (95% CI 0.445–0.653), respectively. The values for the GNRI and albumin levels were comparable for both endpoints (Table S1, Fig. S3), and those for the GNRI and albumin levels were higher than those for BMI (P < 0.01).

We also performed a time-dependent ROC analysis to examine the changes in AUC values for all-cause death and MACE during the follow-up at 3 and 6 months, and 1, 2, 3, and 4 years. The results showed that both the GNRI and albumin levels were strongly associated with all-cause death and MACE throughout the follow-up period, reinforcing their prognostic significance in patients with AMI (Fig. S4).

Overall, these results suggest that the GNRI is a useful prognostic marker in patients with AMI, although it is not superior to albumin values. Previous studies have shown that the GNRI may be superior to albumin levels in the prognostication of patients with cancer and heart failure [31–33], but both indices may be superior in those with AMI.

### Influence of cancer carriers on prognostic value of GNRI

To address the potential impact of cancer, we re-evaluated the prognostic value of the GNRI in a subgroup that excluded cancer carrier patients, who comprised 9.7% of the study population. The results of these analyses showed that the prognostic value of the GNRI remained similar to that obtained in the overall analysis (Fig. S5).

### The potential significance of repeated GNRI measurements

This study evaluated the nutritional status of the patients upon admission. The fact that the results of this study were solely based on the nutritional assessment at admission is crucial. In other words, it is surprising that such significant results were obtained with just a single-point GNRI score at admission. This suggests that the availability of the GNRI at discharge may further enhance the prognostic utility of the GNRI. In addition, including the GNRI score during outpatient visits may increase its utility. While it is important to examine how the prognosis of AMI changes with multiple GNRI measurements, this should be considered in future studies.

### Limitation

This study has several limitations. First, this was a single-center retrospective study. Second, we only considered the GNRI, and it may be important to consider other nutritional indicators. Third, we could not directly compare the present and previous findings from other cohorts with ischemic heart disease, which may limit the generalizability of the present findings to other populations and settings. Fourth, the GNRI, which defines BMI of 22 as indicative of an ideal body weight, is a simple nutritional risk assessment tool, but its utility may be controversial in different populations. Finally, this study involved AMI patients of all ages rather than involving older adults only.

## Conclusion

Time-dependent ROC analysis showed that the GNRI is important in predicting the prognosis of patients with AMI. The optimal cut-off value and reliability of the GNRI altered with changes in the endpoint and observation period. Overall, even a relatively low GNRI at admission was shown to be effective in preventing short-term cardiovascular events. However, a high GNRI score was required to prevent long-term cardiovascular events.

## Supplementary Information

Below is the link to the electronic supplementary material.

Supplementary file1 (PDF 628 KB)

## Data Availability

The datasets generated or analyzed during the current study are available from the corresponding author on reasonable request.

## Abbreviations

GNRI: Geriatric Nutrition Risk Index
BMI: Body mass index
IHD: Ischemic heart disease
AMI: Acute myocardial infarction
CAD: Coronary artery disease
ROC: Receiver operating characteristic
AOC: Area under the curve
CK: Creatine kinase
MACE: Major adverse cardiac events
SD: Standard deviation
