# Supplementary material for: Cut-off values of Geriatric Nutritional Risk Index for cardiovascular events in Japanese patients with acute myocardial infarction
Source: Heart Vessels. 2024 Sep 13;40(3):191–202. doi: 10.1007/s00380-024-02455-w (PMC11846735; doi:10.1007/s00380-024-02455-w)
Supplement: Supplementary file 1 — Supplementary file1 (PDF 628 KB) [file 380_2024_2455_MOESM1_ESM.pdf]

Cut-off values of Geriatric Nutritional Risk Index for cardiovascular events in Japanese patients with acute myocardial infarction

Journal name: Heart and Vessels

Satoshi Ito[1*], Yasunori Inoue[1], Tomohisa Nagoshi[1], Takatoku Aizawa[1], Yusuke Kashiwagi[1], Satoshi Morimoto[1], Kazuo Ogawa[1], Kosuke Minai[1], Takayuki Ogawa[1], Michihiro Yoshimura[1]

[1] Division of Cardiology, Department of Internal Medicine, The Jikei University School of Medicine, Tokyo, JAPAN

*** Corresponding author**

Satoshi Ito, M.D., PhD

Division of Cardiology, Department of Internal Medicine, The Jikei University School of Medicine

3-25-8 Nishi-Shinbashi, Minato-ku, Tokyo, 105-8461, JAPAN

Tel: +81-3-3433-1111 (ex.3261)

Fax: +81-3-3459-6043

E-mail: itousa@jikei.ac.jp

Table S1. Harrell's C-index values derived in the Cox proportional hazard models for all-cause death and MACE.

(A) Endpoint: all-cause death

Predictive models	C-index (95% CI)
GNRI	0.857 (0.786-0.928)
Alb	0.869 (0.802-0.936)
BMI	0.588 (0.460-0.715)

(B) Endpoint: MACE

Predictive models	C-index (95% CI)
GNRI	0.827 (0.762-0.892)
Alb	0.834 (0.771-0.897)
BMI	0.549 (0.445-0.653)

GNRI, Geriatric Nutritional Risk Index; Alb, albumin; BMI, body mass index; MACE, major adverse cardiac events；CI, confidence interval.

Figure S1. Changes over time in the AUC for all-cause death and MACE determined through time-dependent ROC analysis, with the exclusion of patients undergoing hemodialysis.

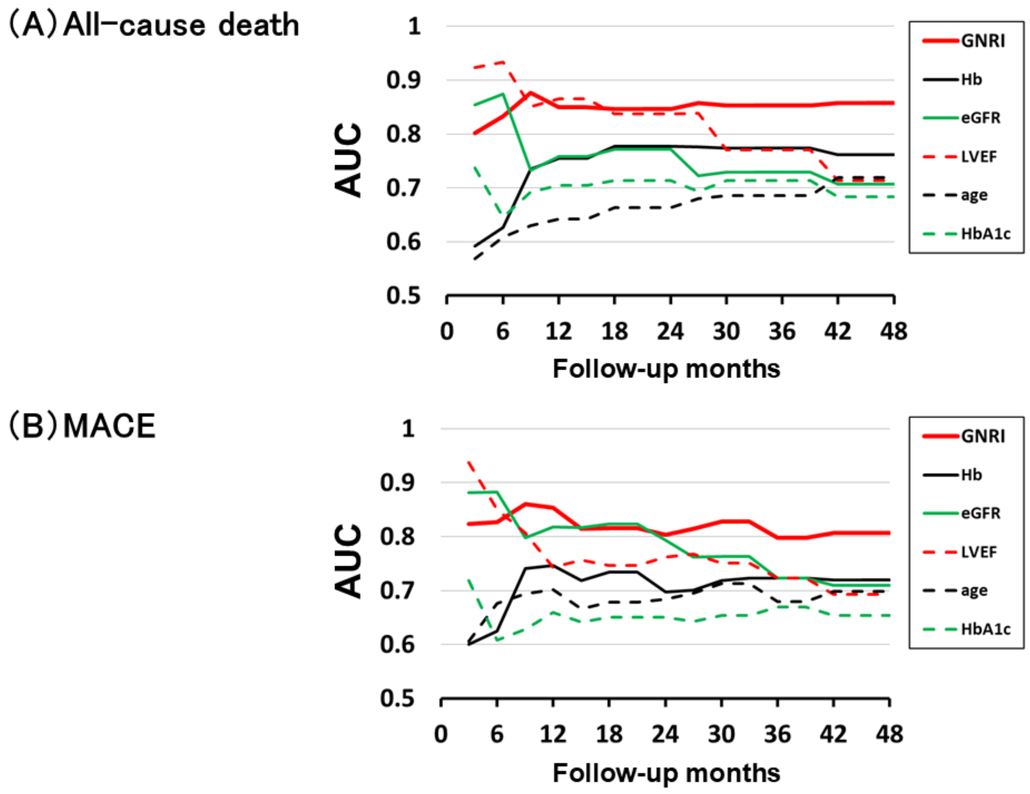

(A) This figure shows the AUC of several markers (GNRI, Hb, eGFR, LVEF, age, and HbA1c) for all-cause death from the start of follow-up, excluding patients undergoing hemodialysis. (B) This figure shows the AUC of several markers (GNRI, Hb, eGFR, LVEF, age, and HbA1c) for MACE from the start of follow-up, excluding patients undergoing hemodialysis. ROC, receiver operating characteristic; GNRI, Geriatric Nutritional Risk Index; Hb, hemoglobin; eGFR, estimated glomerular filtration rate; LVEF, left ventricular ejection fraction; HbA1c, glycated hemoglobin; MACE, major adverse cardiac events; AUC, area under the curve.

Figure S2. Changes over time in the cut-off value for all-cause death and MACE determined through time-dependent ROC analysis, with the exclusion of patients undergoing hemodialysis.

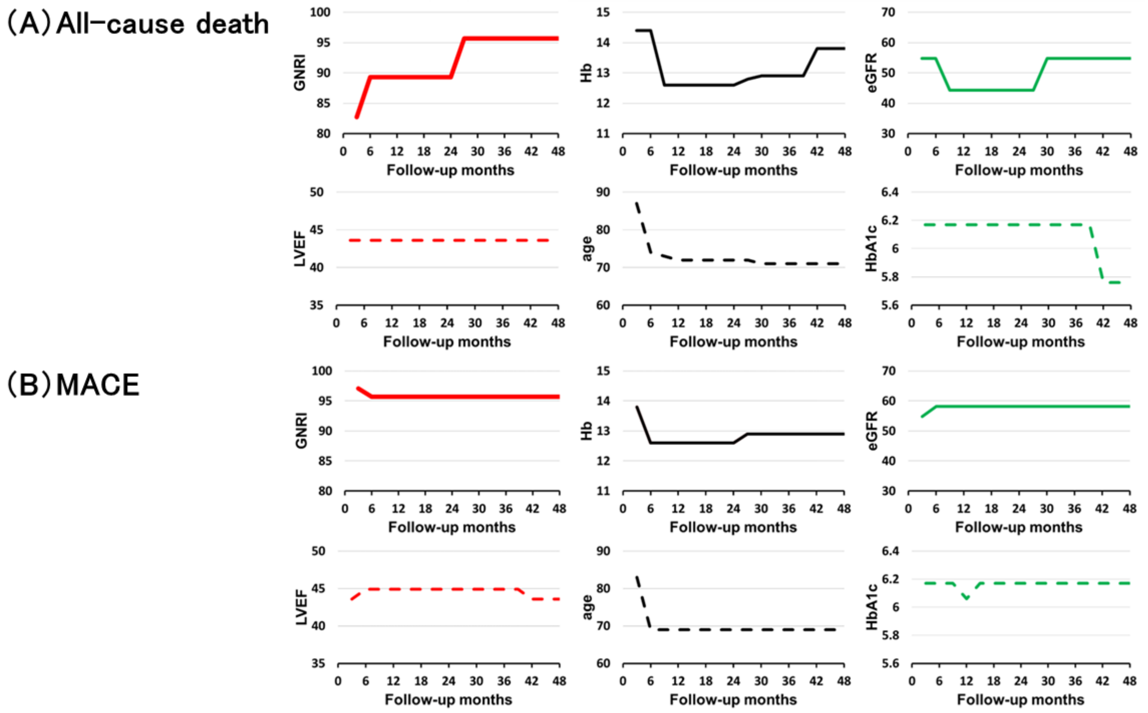

(A) This figure shows the changes over time in the cut-off values of several markers (GNRI, Hb, eGFR, LVEF, age, and HbA1c) for all-cause death from the start of follow-up, excluding patients undergoing hemodialysis. (B) This figure shows the changes over time in the cut-off values of several markers (GNRI, Hb, eGFR, LVEF, age, and HbA1c) for MACE from the start of follow-up, excluding patients undergoing hemodialysis. ROC, receiver operating characteristic; GNRI, Geriatric Nutritional Risk Index; Hb, hemoglobin; eGFR, estimated glomerular filtration rate; LVEF, left ventricular ejection fraction; HbA1c, glycated hemoglobin; MACE, major adverse cardiac events.

Figure S3. Comparison of C-index for GNRI, albumin levels, and BMI for all-cause death and MACE.

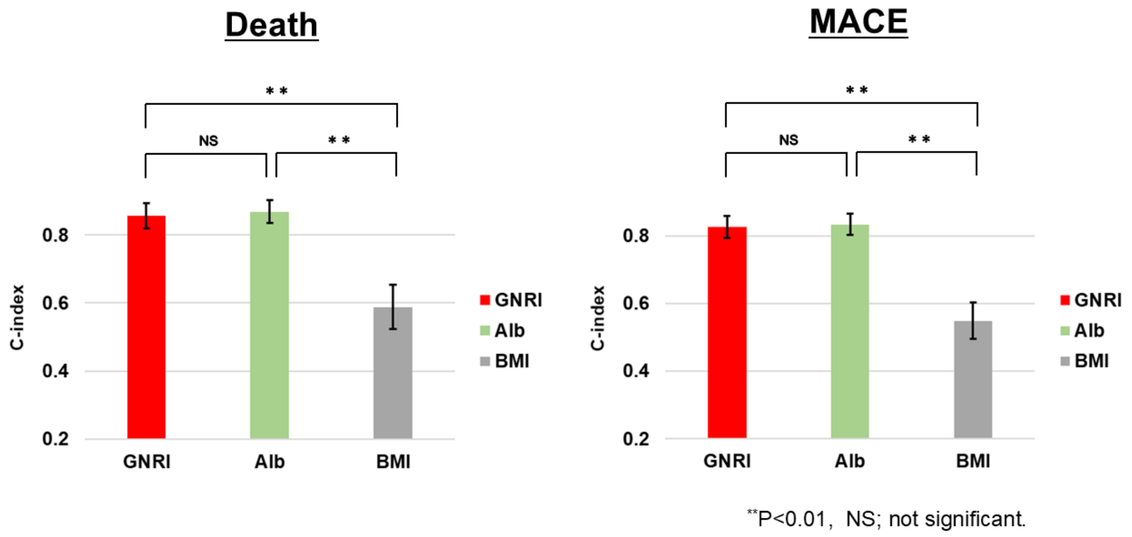

GNRI, Geriatric Nutritional Risk Index; MACE, major adverse cardiac events. **P<0.01, NS; not significant.

Figure S4. Changes over time in the AUC of the GNRI, albumin levels, and BMI for all-cause death (A) and MACE (B) determined through time-dependent ROC analysis.

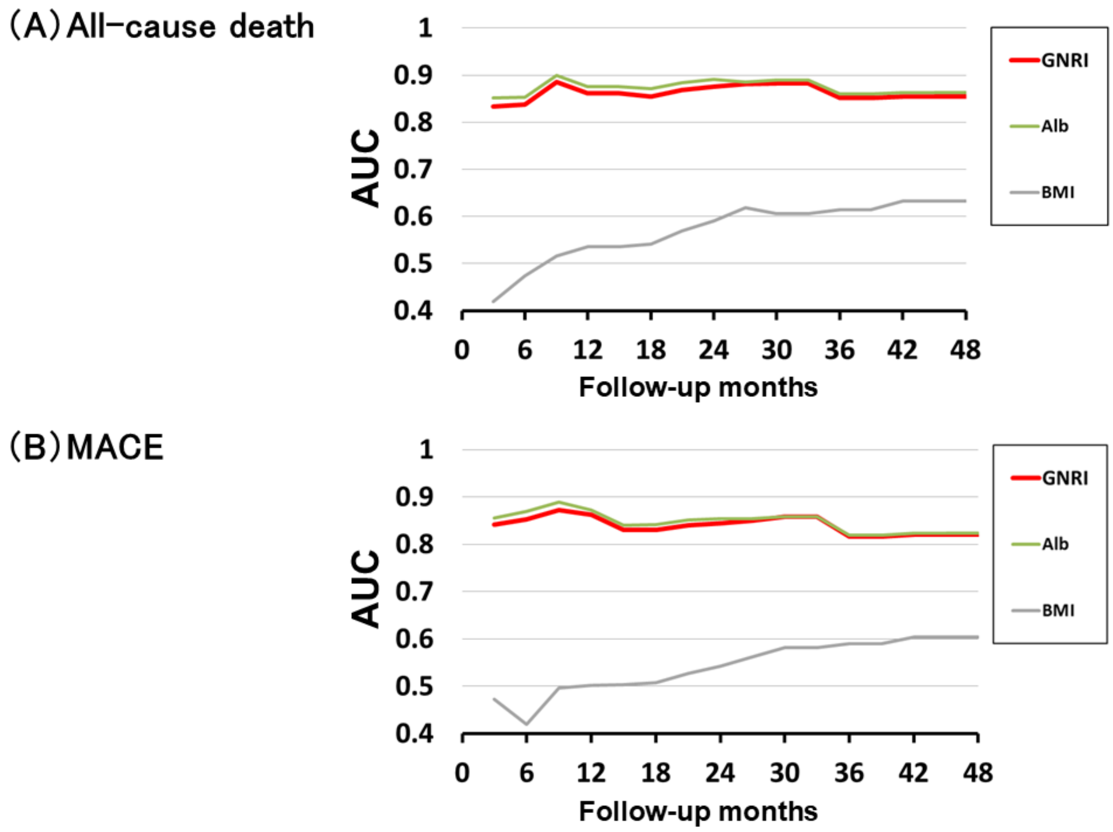

ROC, receiver operating characteristic; GNRI, Geriatric Nutritional Risk Index; MACE, major adverse cardiac events; AUC, area under the curve.

Figure S5. Time-dependent ROC analysis of GNRI for all-cause death and MACE excluding cancer carrier patients.

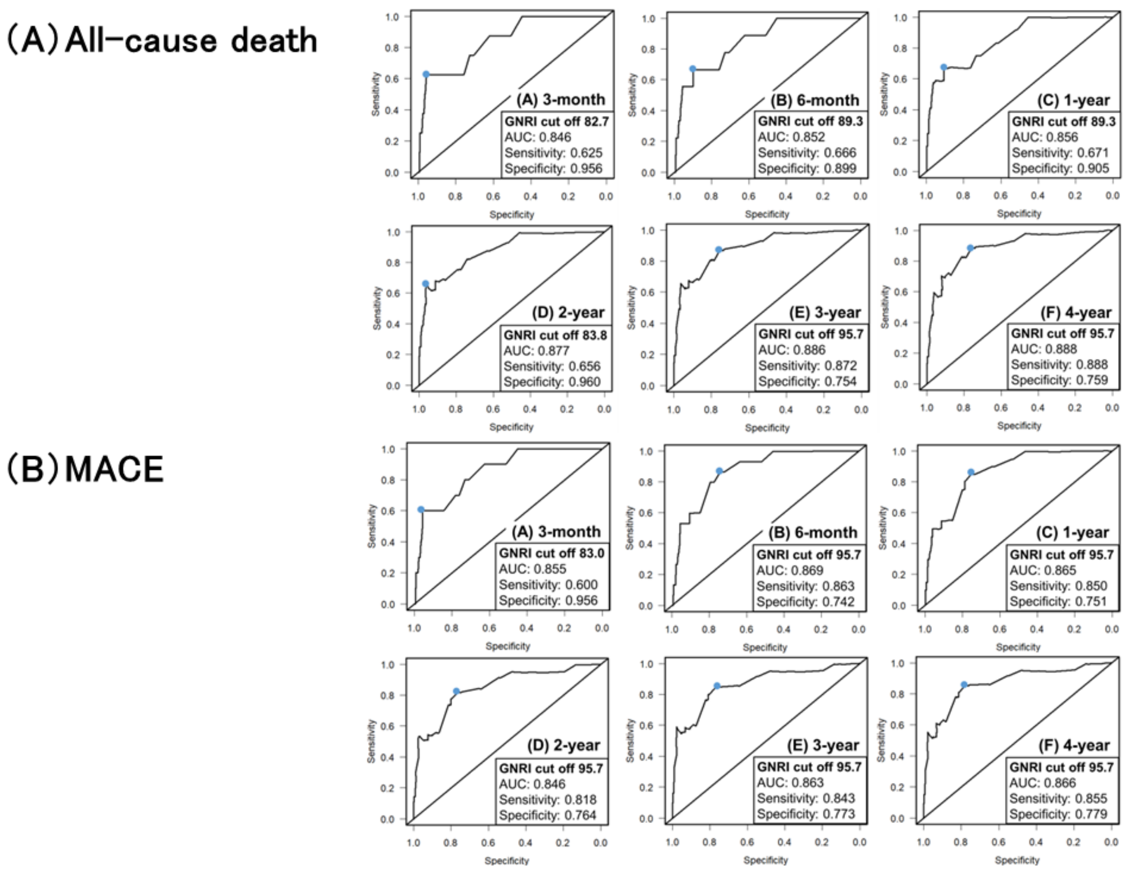

(A) Time-dependent ROC curve analysis of all-cause death excluding cancer carrier patients at (A) 3-month, (B) 6-month, (C) 1-year, (D) 2-year, (E) 3-year, and (F) 4-year.

(B) Time-dependent ROC analysis of MACE excluding cancer carrier patients at (A) 3-month, (B) 6-month, (C) 1-year, (D) 2-year, (E) 3-year, and (F) 4-year. AUC, area under the curve; GNRI, Geriatric Nutritional Risk Index; MACE, major adverse cardiac event; ROC, receiver operating characteristic.
